# High Rates of Bacteremia and Fluoroquinolone Resistance During an Outbreak of Shigellosis Among People Experiencing Homelessness and Opioid Use Disorder in Philadelphia, Pennsylvania

**DOI:** 10.1093/ofid/ofaf296

**Published:** 2025-05-16

**Authors:** Eleanor Stedman, Andrea Molin, Valencia Oglesby, Erin Torpey, Stephanie Spivack, Kaede V Sullivan, Sara Schultz

**Affiliations:** Division of Infectious Disease, Temple University Hospital, Philadelphia, Pennsylvania, USA; Division of Infectious Disease, Temple University Hospital, Philadelphia, Pennsylvania, USA; Division of Infectious Disease, Temple University Hospital, Philadelphia, Pennsylvania, USA; Division of Infectious Disease, Temple University Hospital, Philadelphia, Pennsylvania, USA; Division of Infectious Disease, Temple University Hospital, Philadelphia, Pennsylvania, USA; Department of Pathology and Laboratory Medicine, Temple University Hospital, Philadelphia, Pennsylvania, USA; Division of Infectious Disease, Temple University Hospital, Philadelphia, Pennsylvania, USA

**Keywords:** antibiotic resistance, dysentery, homelessness, opioid use disorder, shigellosis

## Abstract

In 2023, Philadelphia reported an outbreak of *Shigella flexneri* infections. We evaluated all patients hospitalized in our health system with shigellosis during this outbreak. Sixty-seven patients were hospitalized, and 21 (31.3%) were bacteremic. Trimethoprim-sulfamethoxazole was the only antibiotic with reliable susceptibility. Most patients had housing insecurity and opioid use disorder.

## Graphical Abstract

**Graphical Abstract:**
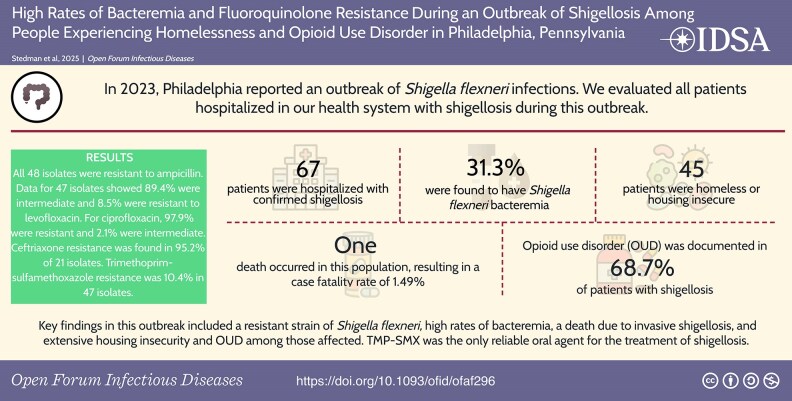
This graphical abstract is also available at Tidbit: https://tidbitapp.io/tidbits/high-rates-of-bacteremia-and-fluoroquinolone-resistance-during-an-outbreak-of-shigellosis-among-people-experiencing-homelessness-and-opioid-use-disorder-in-philadelphia-pa-stedman-et-al-2025-open-forum-infectious-diseases-logo-000000-in-202?utm_campaign=tidbitlinkshare&utm_source=ITP

Invasive shigellosis due to infection with *Shigella* species is a public health concern both nationally and internationally. Transmitted via the fecal-oral route, severe disease typically manifests as profound dysentery causing dehydration and electrolyte derangements [1]. Complications include secondary organ failure, shock, postinfectious arthritis, and rarely hemolytic uremic syndrome [2]. In the United States, *Shigella flexneri* and *Shigella sonnei* predominate, whereas internationally *Shigella dysenteriae* has caused the largest outbreaks of the 20th century [1]. Historically in the United States, outbreaks of shigellosis have been linked to contaminated food and water sources, homeless encampments, and sexual transmission via oral-anal intercourse, particularly among men who have sex with men (MSM) [1, 3, 4]. A recent outbreak of multidrug-resistant *Shigella sonnei* in Vancouver, Canada—disproportionately impacting persons experiencing homelessness (PEH) between 2020 and 2022—revealed a bacteremia rate of 8%, significantly higher than the historical norm of less than 1% typically observed with *Shigella* species [4, 5].

In the fall of 2023, an outbreak of dysentery was reported in the city of Philadelphia, Pennsylvania [5]. The causative agent of the outbreak was identified as *S flexneri* type III. Per department of public health reporting, the outbreak predominantly affected people with opioid use disorder (OUD) and PEH [6]. This study involved an expanded investigation of the epidemiology of cases presenting to Temple University Health System (TUHS), a health system that serves North Philadelphia, an impoverished community with a high burden of human immunodeficiency virus (HIV), hepatitis C, OUD, and homelessness.

## METHODS

To characterize the outbreak in our health system, a retrospective study was conducted. All patients hospitalized at TUHS with confirmed shigellosis between 1 October 2023 and 30 April 2024 were included in the study. Confirmed shigellosis was defined as growth of *Shigella* species in stool or blood culture, or positive testing for *Shigella* by real-time polymerase chain reaction (PCR) via a send-out stool pathogen panel. The stool pathogen panel identified *Shigella* to the genus level only. All *Shigella* isolates that were recovered from culture were sent to the Pennsylvania State Public Health Laboratory, which confirmed organism identification to the species level and determined the serotype. The medical records of patients with confirmed shigellosis during the study window were reviewed for patient age, sex, housing status, history of OUD and injection behavior based on history obtained during clinician assessment, HIV status, stool and blood culture data, stool pathogen panel data, antibiotic susceptibility data, and death during hospitalization. The Temple University Institutional Review Board approved the study.

## RESULTS

**Figure 1.**
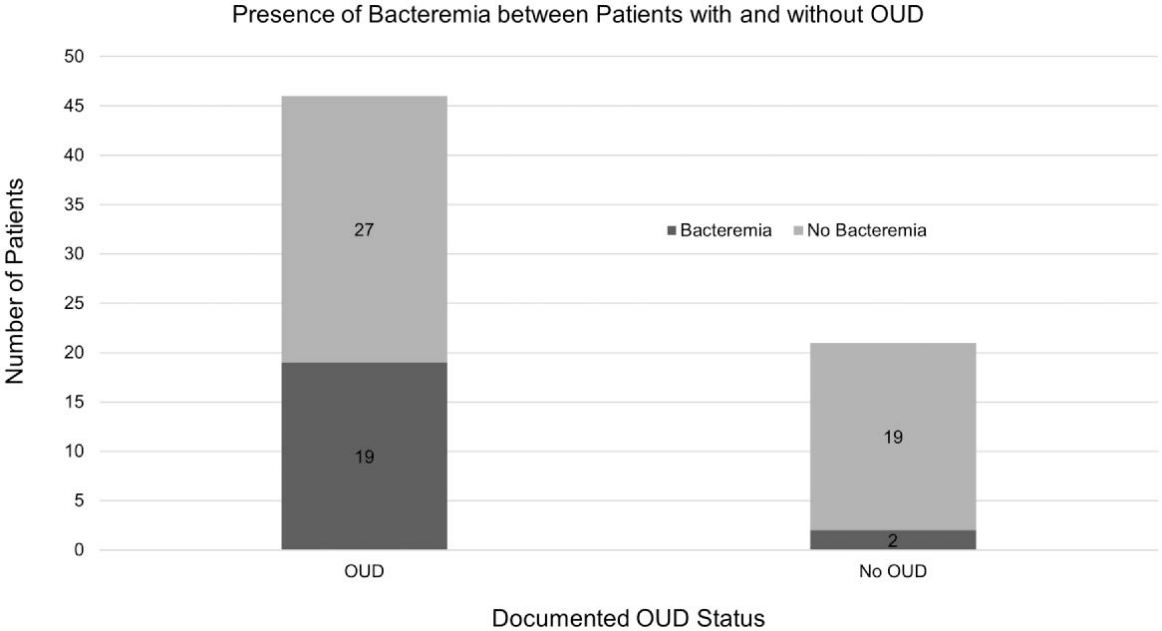
Comparative cases of *Shigella flexneri* bacteremia between patients with and without documented opioid use disorder (OUD).

In total, 67 patients were hospitalized at TUHS from 1 October 2023 to 30 April 2024 with confirmed shigellosis. The median age of hospitalized patients was 41 years (interquartile range, 32–51 years). There was a slight sex imbalance, with 60% of patients being male. Forty-five patients (67%) were documented to be experiencing homelessness or housing insecurity, and OUD was documented in 46 (68.7%) patients with shigellosis. Of those with OUD, 35 patients (76.1%) exhibited injection behavior. HIV infection was documented in 3 patients (4%), while 51 patients (76%) tested negative for HIV. An additional 13 patients (19%) had no documented HIV testing in our electronic medical record system. Twenty-four patients (35.8%) were diagnosed with shigellosis via a positive stool culture, and 19 patients (28.3%) were diagnosed via a PCR-based stool pathogen panel alone. Of the 48 cases in which *Shigella* was recovered from culture, 42 (88%) were confirmed as *S flexneri* type III. The other cases were *S flexneri* type I (n = 1), type II (n = 3), and variant X (n = 2). Eight patients had >1 positive *Shigella* test; 5 had positive blood and stool cultures; and 3 had positive stool culture and stool pathogen panel. The remaining 16 patients (23.9%) were diagnosed from positive blood cultures alone. Bacteremia with *S flexneri* was documented in 21 patients, reflecting a 31.3% rate of bacteremia among hospitalized patients. Stool cultures were not obtained for 4 patients (6%) of the total study population, though the reasons for this omission remain unclear. Notably, all 4 of these patients had *Shigella* bacteremia. Among patients with documented OUD, 19 (41.3%) were bacteremic (Figure 1). Additionally, 1 death occurred in our population, marking a case fatality rate of 1.49%.

**Figure 2.**
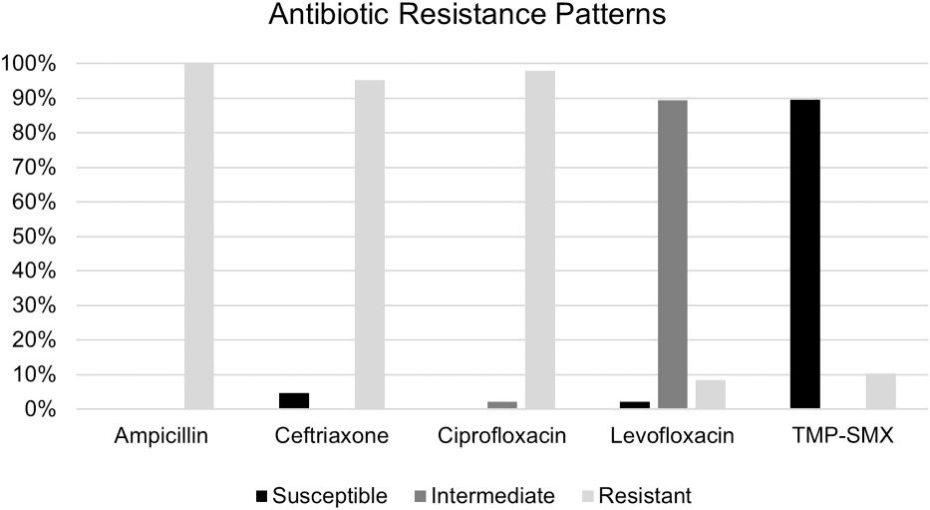
Antibiotic resistance patterns among 48 cultured isolates of *Shigella flexneri.*

Out of 48 cultured isolates, all were resistant to ampicillin. Levofloxacin susceptibility data were available for 47 isolates, with 89.4% showing intermediate resistance and 8.5% being resistant. For ciprofloxacin, susceptibility data were also available for 47 isolates, of which 2.1% were intermediate and 97.9% were resistant. Ceftriaxone susceptibility was assessed in 21 isolates, with 95.2% found to be resistant. Finally, trimethoprim-sulfamethoxazole (TMP-SMX) susceptibility was available for 47 isolates, with 10.4% demonstrating resistance (Figure 2). Carbapenem and macrolide susceptibility was not routinely reported on susceptibility reports.

## DISCUSSION

Notable findings in this outbreak included a particularly multidrug-resistant strain of *S flexneri*, high rates of bacteremia in hospitalized patients, a death due to invasive shigellosis, and extensive housing insecurity and OUD among those affected. Given the near-universal fluoroquinolone and extensive β-lactam resistance, TMP-SMX was the only reliable oral agent for treatment of shigellosis.

The 31.3% overall rate of bacteremia in hospitalized patients is unprecedented in the published literature. Moreover, 7 patients with a positive stool culture or gastrointestinal PCR pathogen panel did not have blood cultures collected, suggesting that our rate of bacteremia may have been underestimated. Furthermore, 1 patient in our cohort died from invasive shigellosis. He presented late to hospital care and was bacteremic, with presumed septic and hypovolemic shock, ultimately leading to death. Temple Health serves an economically disadvantaged population with high burden of comorbid illnesses. As a result, patients often present to our hospital with severe and advanced forms of disease. Given this, it is possible that our study may have been subject to bias toward severe presentations of *Shigella* including bacteremia.

The total number of patients hospitalized with shigellosis may have been higher than 67, as we are aware that many patients were treated empirically during this outbreak. The underdetection of cases was likely due to the limited sensitivity in stool culture [7] and underuse of the stool pathogen panel given delay of results in send-out testing. An additional limitation of this study is the lack of specificity in the PCR stool pathogen panel. As the panel did not identify *Shigella* to the species level, patients with shigellosis diagnosed only with this method may have had non-*flexneri* infections, although we consider this less likely given the extent of the outbreak at the time. Furthermore, due to the small sample size (N = 67), our statistical analysis was restricted to descriptive statistics. A larger study could have enabled univariate and multivariate analyses to better explore the relationship between OUD and *Shigella* bacteremia. One additional limitation is that information on MSM status was not collected, preventing us from evaluating MSM as a potential risk factor for shigellosis in our population.

Most cases of *Shigella* bacteremia in this outbreak occurred in patients with OUD. While we propose several potential explanations for this outcome, the descriptive nature of our study precludes definitive conclusions. It is possible that fecal contamination of hands during injection drug use may have led to direct inoculation of the bloodstream with *S flexneri*, as most of our patients with OUD engage in injection behavior. It is also possible that patients with severe OUD experience malnutrition and undermanagement of chronic medical conditions, and that these patients were therefore at higher risk for severe colitis, which resulted in secondary bacteremia. Most hospitalized patients also had housing insecurity. Patients reported anecdotally that there was diarrheal contamination of many public spaces during this outbreak, reflecting the insufficient sanitation facilities for people experiencing homelessness. This may have contributed to the duration of the outbreak, which extended over many months. Overall, these findings highlight the ongoing risk of infectious diarrheal disease among people with housing insecurity and identify OUD as a new risk factor for invasive shigellosis, particularly *Shigella* bacteremia.

## Notes

***Author contributions.*** E. S. and A. M. contributed to study design, data extraction, data analysis, and manuscript preparation. E. T. and V. O. contributed to study design and manuscript preparation. S. Sc., S. Sp., and K. V. S. contributed to study design, study supervision, and manuscript preparation.

***Acknowledgments.*** We appreciate the collaboration with colleagues at the Philadelphia Department of Public Health and Prevention Point Philadelphia during this outbreak.

***Data availability.*** Data are not publicly available.

***Patient consent.*** This study did not include factors necessitating patient consent. The Temple University Institutional Review Board approved the study.

***Potential conflicts of interest**.* The authors: No reported conflicts of interest.
